# A positive feedback loop between Periostin and TGFβ1 induces and maintains the stemness of hepatocellular carcinoma cells via AP-2α activation

**DOI:** 10.1186/s13046-021-02011-8

**Published:** 2021-06-30

**Authors:** Gang Chen, Yi Wang, Xin Zhao, Xiao-zai Xie, Jun-gang Zhao, Tuo Deng, Zi-yan Chen, Han-bin Chen, Yi-fan Tong, Zhen Yang, Xi-wei Ding, Peng-yi Guo, Hai-tao Yu, Li-jun Wu, Si-na Zhang, Qian-dong Zhu, Jun-jian Li, Yun-feng Shan, Fu-xiang Yu, Zheng-ping Yu, Jing-lin Xia

**Affiliations:** 1 Department of Hepatobiliary Surgery, The First Affiliated Hospital of Wenzhou Medical University, Wenzhou, Zhejiang, 325005 China; 2 Key Laboratory of Diagnosis and Treatment of Severe Hepato-Pancreatic Diseases of Zhejiang Province, The First Affiliated Hospital of Wenzhou Medical University, Wenzhou, 325005 China; 3 Liver Cancer Institute, The First Affiliated Hospital of Wenzhou Medical University, Wenzhou, 325005 China; 4 Division of Preventive Medicine, School of Public Health and Management, Wenzhou Medical University, Wenzhou, 325005 China; 5 Department of General Surgery, The First Affiliated Hospital of Soochow University, Suzhou, 215006 China; 6 Department of Infectious Diseases, Shandong Provincial Hospital affiliated to Shandong University, Jinan, 250021 China; 7 Department of Gastroenterology, Nanjing Drum Tower Hospital, The Affiliated Hospital of Nanjing University Medical School, Nanjing, 210008 Jiangsu China; 8 Liver Cancer Institute, Zhongshan Hospital, Fudan University, Shanghai, 200032 China

**Keywords:** Hepatocellular carcinoma, Cancer stem cells, POSTN, AP-2α, Positive feedback loop

## Abstract

**Background:**

Liver cancer stem cells (LCSCs) play key roles in the metastasis, recurrence, and chemotherapeutic resistance of hepatocellular carcinoma (HCC). Our previous research showed that the *POSTN* gene is closely related to the malignant progression and poor prognosis of HCC. This study aimed to elucidate the role of *POSTN* in generating LCSCs and maintaining their stemness as well as the underlying mechanisms.

**Methods:**

Human HCC tissues and matched adjacent normal tissues were obtained from 110 patients. Immunohistochemistry, western blotting (WB), and RT-PCR were performed to detect the expression of POSTN and stemness factors. The roles of transforming growth factor (TGF)-β1 and AP-2α in the POSTN-induced stemness transformation of HCC cells were explored in vitro and in vivo using LCSCs obtained by CD133^+^ cell sorting.

**Results:**

The high expression of POSTN was correlated with the expression of various stemness factors, particularly CD133, in our HCC patient cohort and in TCGA and ICGC datasets. Knockdown of POSTN expression decreased the abilities of HCC cell lines to form tumours in xenograft mouse models. Knockdown of POSTN expression also suppressed cell viability and clone formation, invasion, and sphere formation abilities in vitro. Knockdown of AP-2α attenuated the generation of CD133^+^ LCSCs and their malignant behaviours, indicating that AP-2α was a critical factor that mediated the POSTN-induced stemness transformation and maintenance of HCC cells. The role of AP-2α was verified by using a specific αvβ3 antagonist, cilengitide, in vitro and in vivo. Activation of POSTN could release TGFβ1 from the extracellular matrix and initiated POSTN/TGFβ1 positive feedback signalling. Furthermore, we found that the combined use of cilengitide and lenvatinib suppressed the growth of HCC cells with high POSTN expression more effectively than the use of lenvatinib alone in the patient-derived xenograft (PDX) mouse model.

**Conclusions:**

The POSTN/TGFβ1 positive feedback pathway regulates the expression of stemness factors and the malignant progression of HCC cells by regulating the transcriptional activation of AP-2α. This pathway may serve as a new target for targeted gene therapy in HCC.

**Supplementary Information:**

The online version contains supplementary material available at 10.1186/s13046-021-02011-8.

## Background

Hepatocellular carcinoma (HCC) is one of the most common malignant tumours, ranking fifth in worldwide incidence and third in mortality among all tumours [1]. Nearly half of the newly diagnosed patients each year are in China, which is a region with a high HCC incidence. With improved surgical technologies and pharmacological therapies, the 5-year survival rate of HCC after radical resection has reached a level higher than 30%. However, tumour metastasis and postoperative recurrence remain major threats that affect the long-term outcomes of HCC patients [2]. In recent years, liver cancer stem cells (LCSCs) have become a hot topic in basic research on HCC. LCSCs are more closely related to HCC metastasis and recurrence than non-LCSCs due to their more robust metastatic and tumorigenic properties. In addition, LCSCs are more tolerant to traditional chemotherapy, which is one reason for the low efficacy of radiotherapy and chemotherapy in liver cancer patients [3]. Therefore, an increasing number of researchers believe that the key to improving the curative effect of HCC treatment is the elimination of LCSCs, and future gene therapy approaches for HCC may shift from killing HCC cells or reducing tumour size alone to eliminating LCSCs.

LCSCs are defined as cell clusters with surfaces enriched in markers including EpCAM, CD133, CD13, CD90, CD44, CD24, and calcium channel a2d1 subunit [4–6]. Since the concept of cancer stem cells (CSCs) was proposed, the origin of these CSCs has been controversial. Most researchers believe that the CSCs in many tumours, including HCC, are derived from the abnormal proliferation and differentiation of normal stem cells [7]. Other studies have argued that CSCs could also be produced by differentiated mature tumour cells in response to induction factors [8–10]. Tumour microenvironment components also play essential roles in transforming tumour cells into tumour stem cells [11, 12]. However, the question of how LCSCs form and sustain their self-renewal abilities remains to be addressed. Our previous research confirmed that Periostin (*POSTN*) is a crucial gene involved in the angiogenesis and pulmonary metastasis of HCC; it is highly expressed in 60% of liver cancer patients, and its high expression indicates poor prognosis [13]. *POSTN* gene expression is upregulated in most common tumours, including breast cancer, pancreatic cancer, melanoma, and colon cancer, and it plays an important role in the development of tumours, especially in the metastasis of tumours [14]. Recent studies have shown that the POSTN protein plays a vital role in the formation and maintenance of the stemness-enhancing microenvironment of tumours, thus promoting tumour metastasis and invasion [15]. Ouyang G. recently confirmed that exogenous POSTN protein can induce breast epithelial cells and breast cells to acquire a stem cell phenotype [16]. Lambert et al. found that in the tumour microenvironment, the POSTN protein is a factor that is important for the maintenance of breast cancer stem cells [17]. Recent research also revealed that when the POSTN protein is released from activated hepatic stellate cells, it promotes the acquisition of a stem cell phenotype in the surrounding live cancer cells. Based on the research findings described above, one can see that the upregulation of POSTN expression in HCC might be closely related to the formation and maintenance of LCSCs, but the underlying mechanism remains unclear.

In this study, we found that the *POSTN* gene was closely related to stemness markers in HCC. POSTN formed a positive feedback loop with TGFβ1 to induce and maintain its high expression in the tumour microenvironment. AP-2α was identified as a critical transcription factor for regulating CD133 gene expression in HCC cells, and AP-2α promoted the POSTN-induced transformation of HCC cells into LCSCs and maintained their stemness. Therefore, the POSTN/TGFβ1/AP-2α pathway may be a new target to bring a breakthrough for the targeted therapy of HCC.

## Materials and methods

A comprehensive list of all the reagents, kits, antibodies, and primers used in this study can be found in Supplementary Tables S1 and S2.

### Patients and tissues preparation

Specimens, including tumour tissues and matched adjacent normal tissues (≥2 cm from the resection margin), were collected from 110 HCC patients who underwent surgical resection in the Department of Hepatobiliary Surgery, The First Affiliated Hospital of Wenzhou Medical University, between November 2008 and October 2010. The HCC patients were between 24 and 79 years old (median age was 52 years old), and the median follow-up time was 38.2 months. The clinicopathological features analysed in this study included postoperative tumour-node-metastasis (TNM) staging and Edmondson-Steiner grading by experienced pathologists. In addition, another batch of liver cancer and adjacent tissue samples were obtained between November 2017 and March 2018. Each of these samples was divided into three parts: one was used to establish a PDX model, one was flash frozen and stored at − 80 °C for molecular analysis, and one was fixed in formaldehyde for histopathological examination. This study was approved by the ethics committee of The First Affiliated Hospital of Wenzhou Medical University, and written informed consent was obtained from each patient.

### Bioinformatics analysis

The mRNA expression data of 349 HCC specimens from The Cancer Genome Atlas (TCGA) database and 240 HCC specimens from the International Cancer Genome Consortium (ICGC) database were extracted for analysis. The *Phatmap* package and the *corolot* package in R language were used to generate the correlation matrix heat map between the *POSTN* gene and stemness-related factors.

The GSE1898 dataset includes data of HCC tumour tissues and matched benign tumour-adjacent tissues obtained from 139 individuals (61 Chinese patients and 78 Caucasian patients) by surgical resection. The details of the microarray gene expression profile were reported by Lee JS et al. [18]. The fold change (FC) in the POSTN transcript was calculated as the ratio of the gene expression value in tumour tissues to that in normal tissues, and the mean value in normal tissues was used as the baseline. A log_2_-transformed tumour/normal value ≥1.0 (i.e., a fold change of tumour vs. normal ≥2) or ≤ − 1.0 indicated an increase or decrease in gene expression, respectively. Spearman rank correlation analysis was used to analyse the correlation between POSTN and stemness-related factors.

Transcriptome sequencing of HCC cell lines with different levels of *POSTN* gene expression was conducted by GeneChem Biotechnology Co., Ltd., Shanghai (GSAE0154536). The change in the POSTN transcript copy number was calculated based on the ratio of the expression level in tumour tissues to that in normal tissues, and the median value in normal tissues was used as the baseline. The Venn diagram shows the genes associated with the top 50 upregulated genes in Hep3B cells after transfection with control and p-POSTN expression plasmids, the genes associated with the top 50 downregulated genes in HCCLM3 cells after transfection with lentivirus carrying shRNA targeting POSTN and matched scrambled RNA, and 19 genes encoding transcriptional regulators of CD133 as predicted by the TRANSFAC transcription factor prediction database.

### Establishment of orthotopic and subcutaneous xenograft mouse models

All the animal experiments were approved by the Wenzhou Medical University Animal Care and Use Committee. A total of 48 BALB/c nude mice (Laboratory Animal Center, Wenzhou Medical University, Wenzhou, China) were randomly divided into eight groups with six mice in each group, including four groups that underwent orthotopic implantation of liver cancer cells (Hep3B *p*-cDNA3.1, Hep3B *p*-POSTN, LM3 Scr-POSTN, and LM3 Sh-POSTN) and four groups that underwent subcutaneous implantation of tumours. After the mice were anaesthetized, laparotomy was conducted to expose the left lobe of the liver for the orthotopic implantation of liver cancer cells carrying a luciferase reporter gene. After the operation, the growth status of the mice was evaluated weekly, and the fluorescence intensity was measured regularly. For imaging, the mice were intraperitoneally injected with the D-luciferin substrate (15 mg/mL) at a dose of 10 μL/g; 10–15 min after injection, imaging was performed on the IVIS-SPECTRUM in vivo imaging system (PerkinElmer). The images were analysed, and the fluorescence intensity values after tumour cell inoculation were measured by using in vivo imaging software (Caliper Life Sciences). To establish a subcutaneously transplanted tumour model, the mice were subcutaneously implanted with liver cancer cells in the lateral side of the right hind limb. Tumour growth was monitored by measuring the tumour size twice a week with a Vernier caliper. On the 33rd day after xenograft establishment, all the mice were sacrificed to harvest subcutaneous tumour, lung, and brain tissue samples. The volume of the tumour was determined by using the formula: volume = width×length× 0.52. All three tissue types were fixed with 4% paraformaldehyde and embedded in paraffin for subsequent IHC analysis. All the animal experiments were strictly conducted according to the Guide for the Care and Use of Laboratory Animals, and prior to the animal experiments, an agreement was drafted based on the 1975 Declaration of Helsinki and approved by the Animal Ethics and Welfare Committee of Wenzhou Medical University.

### PDX model preparation and drug tests

Fresh tumour tissues extracted from patients in the operating room were cut into small blocks approximately 1 mm^3^ in size under aseptic conditions. The nude mice were anaesthetized with isoflurane and maintained in the anaesthetic state. In a sterile environment, a skin incision approximately 0.5 cm long was made on the lateral side of the right hind limb using a sterilized apparatus. The small human tumour tissue blocks were then subcutaneously implanted into the mice, and the skin incision was closed. Tumour growth was monitored regularly by measuring the size of the tumours twice a week with a Vernier caliper. When the tumours grew to approximately 200 mm^3^, the mice were given intragastric administration of lenvatinib (10 mg/kg) and intraperitoneal injection of cilengitide (20 mg/kg) every 3 days. The body weight and tumour growth of the mice were monitored.

### Tissue microarray and immunohistochemical (IHC) analysis

Following the previously described IHC staining protocol [19], tumour tissues and the corresponding adjacent normal tissues were fixed in 4% paraformaldehyde immediately after harvest. After fixation, the tissues were subjected to a series of procedures, including paraffin embedding, sectioning (thickness of 4 μm), hydration in different concentrations of ethanol after dimethyl benzene dewaxing, antigen repair after washing (boiling in 1× citric acid-disodium hydrogen phosphate buffer), and blocking of the endogenous catalase activity. Then, the sections were stained with antibodies against CD90, CD133, CK19, TWIST, SNAIL, CXCR4, and αSMA. Negative controls were established by substituting the primary antibody solutions with 1% bovine serum albumin (BSA)/Tris-buffered saline (TBS). Finally, the slides were cover slipped and observed under a microscope.

To semi-quantitatively assess protein expression, the IHC staining intensity was scored from 0 to 3, with 0, 1, 2 and 3 indicating no staining and weak, medium and strong staining, respectively. In addition, the percentage of positive cells was scored as 0 points for less than 5%, 1 point for 6–25%, 2 points for 26–50%, 3 points for 51–75%, and 4 points for above 75%. The final staining score was determined by multiplying the staining intensity score by the corresponding positive-cell percentage point. The microvascular density (MVD) was calculated by counting the CD31-positive cells, which were identified by a brown precipitate in the cytoplasm of endothelial cells; the vessels in each section were counted in 5 microscopic fields [20].

### Flow cytometry (FCM)

To determine the proportion of CD133^+^ cells among hepatocellular carcinoma cells, cells were harvested, washed twice with PBS, resuspended in cell staining buffer at a concentration of 5 × 10^5^/100 μl and incubated on ice for 30 min in the dark with FITC-conjugated antibodies against CD133 at a dilution of 1:200. After washing, the cells were sorted by using a BD FACSCalibur. Follow-up analysis was performed using FlowJo Software (Ashland, Kentucky, USA). Positive and negative gates were determined using immunoglobulin G (IgG)-stained and unstained controls.

### Polymerase chain reaction (PCR)

Total RNA was extracted from cells and HCC specimens using TRIzol reagent and was used for cDNA synthesis after its concentration was determined. Reverse transcription was conducted following the instructions provided in the RevertAid First-Strand cDNA Synthesis Kit. Subsequently, the synthesized cDNA was amplified using SYBR Premix Ex Taq (Perfect Real Time) in an ABI 7300 Real-Time System. The mRNA expression level of each candidate transcription factor was measured and normalized to the expression of 18S ribosomal RNA as an internal reference. The reaction conditions were as follows: 95 °C for 10 min, followed by 40 cycles of 95 °C for 15 s and 60 °C for 60 s. The primer sequences are listed in Supplementary Table S2.

### Western blotting (WB) and co-immunoprecipitation (co-IP)

Cell or tissue samples were lysed in lysis solution, and the total protein concentration was determined by using the bicinchoninic acid (BCA) kit. The samples were loaded into 4–15% sodium dodecyl sulfate - polyacrylamide gel electrophoresis (SDS− PAGE) gels (30 μg of protein per lane) for electrophoresis, and then, the proteins were transferred onto polyvinylidene fluoride (PVDF) membranes. Next, the membranes were incubated with 5% BSA for 1 h to block nonspecific signals and then washed with TBST [10 mM tris (pH 8.0), 150 mM NaCl, and 0.05% Tween 20] three times. Then, the membranes were incubated with primary antibodies against POSTN, FOXP3, c-Myc, AP-2α, CD133, avβ3, TGFβ1, β-actin, or GAPDH overnight at 4 °C. The concentrations of the antibodies are listed in Supplementary Table S1. After being thoroughly washed three times with TBST, the membranes were incubated with secondary goat anti-rabbit or goat anti-rat antibodies coupled to horseradish peroxidase at room temperature for 1 h, followed by three washes with TBST. Finally, the immune complexes were detected using a HyGLO HRP assay kit and analysed by a gel imaging analysis system. β-actin used as the internal control.

After washing three times using PBS with Triton X-100 (PBST), magnetic beads were incubated with anti-avβ3, anti-TGFβ1 and IgG antibodies on a shaker at room temperature for 1 h and then washed three times with PBST. Next, the antibody-coupled beads were incubated with cellular protein lysates overnight at 4 °C. After the supernatant was removed, the beads were washed with PBST three times and incubated with diluted elution buffer at 70 °C for 10 min. Finally, the pulled down protein complexes were analysed by SDS-PAGE/immunoblotting analysis.

### ChIP experiment

After crosslinking with 1% formaldehyde, the cells were lysed, and the DNA was broken into approximately 600-bp fragments using a Bioruptor 300 (Diagenode). Subsequently, immunoprecipitation was performed using magnetic beads and POSTN antibodies or normal rabbit IgG antibodies to bind the cleaved chromatin. After reversing the formaldehyde cross-linking of the protein and DNA complexes, the immunoprecipitated DNA was purified by using a ChIP column. The identity and quantity of the isolated DNA fragments were determined by PCR using primers designed to amplify the POSTN promoter region, which potentially contained CD133 binding sites. The primer sequences are listed in Supplementary Table S2. A C1000 Thermal Cycler (Bio-Rad) was used for PCR quantification, and each experiment was repeated three times.

### Double immunofluorescence (IF) staining

Cells were washed with Dulbecco’s PBS (D-PBS) and fixed with N,N′-dipiperazine containing 2.5% formaldehyde at room temperature for 20 min. After fixation, the cells were washed with D-PBS and then blocked with 5% goat serum at 37 °C for 1 h. Next, the cells were incubated with primary antibodies against POSTN, AP-2α, CD133, or TGFβ1 at 37 °C for 1 h. After washing three times with D-PBS, the cells were then incubated with Alexa Fluor 488-conjugated anti-mouse IgG and Alexa Fluor 594-conjugated anti-rabbit IgG in the dark for 1 h. After three washes, the cells were incubated with 4′-6-diamidino-2-phenylindole (DAPI) for 1 min, sealed with Prolong Gold anti-fading reagent, and observed under a confocal microscope. In the negative control group, PBS was used to replace the primary antibodies.

### Dual-luciferase reporter assay

Dual-luciferase reporter plasmids, in which the luciferase reporter gene was inserted downstream of the CD133 promoter region, were synthesized by GeneChem Biotechnology Co., Ltd. The cells were seeded in 24-well plates; when the cells reached approximately 80% confluence, they were co-transfected with plasmids containing the Renilla luciferase gene and reporter gene with Lipofectamine™ 3000 for 48 h following the instructions. After the medium was discarded, the cells were washed twice with PBS and then lysed in 100 μl of Passive Lysis Buffer (PLB) on a shaker at room temperature for 30 min. Next, 20 μl of the cell lysates was transferred to an EP tube and gently mixed with 50 μl of luciferase assay reagent II (LAR II) using a pipette to avoid generating bubbles. The fluorescence assay was performed using a GloMax 20/20 Luminometer (Promega). After detection, the EP tube was removed, and the reaction was quenched by adding 50 μl of Stop & Glo Reagent. The fluorescence intensity of the internal reference, Renilla luciferase, was measured again.

### Cell proliferation analysis (CCK-8 assay)

Cells, including LM3 cells, SNU-387 cells transfected with short hairpin RNA (shRNA) or scrambled (scrRNA) lentiviruses; Hep3B cells, PRF5 cells transfected with *p*-POSTN or *p*-cDNA3.1 overexpressed plasmid, were seeded in 96-well plates (5000 cells/well). After 6 h of incubation, the cells were washed with PBS three times. Fresh culture medium containing 0.5% FBS was added. Cell proliferation was assessed by the Cell Counting Kit-8 cell proliferation assay kit, and the absorbance was measured at a wavelength of 450 nm. Each experiment was repeated three times.

### Cell colony formation assay

Cells were seeded in six-well plates at a density of 5 × 10^3^ cells per well, and the medium was changed every 3 days. After 2 weeks of culture, the medium was removed, and the cells were washed with PBS three times to remove the unbound cells. The adherent cells were fixed with 4% paraformaldehyde at room temperature for 30 min and then stained with 0.01% crystal violet for 30 min. Cell colonies containing > 50 cells were counted. The experiment was repeated three times.

### Cell invasion assay

The cells were seeded in Transwell inserts containing Matrigel-coated porous membranes (1 × 10^4^ cells per insert) and cultured in serum-free DMEM. Complete medium containing 10% FBS was added to the lower chamber. After 12 h of incubation, the cells remaining in the insert were removed with cotton swabs. The cells that were adherent to the lower side of the membrane were fixed with 10% acetic acid/90% methanol and subjected to haematoxylin and eosin (H&E) staining. For each Transwell membrane, five visual fields with the most migrated cells were selected, and the cells were counted under a microscope at 400× magnification. The experiment was repeated three times.

### Cell sphere formation assay

The cells in each group were evenly seeded into a low-viscosity 96-well plate (500 cells per well) and cultured in DMEM/F12 medium (1:1) containing B27 supplement, antibiotics, epidermal growth factor, basic fibroblast growth factor, hepatocyte growth factor, and 1% methylcellulose. The cells were incubated at 37 °C, and the culture medium was changed every 3 days. After 14 days of culture, spheres with a diameter > 75 μm were counted.

### Statistical analysis

The data are expressed as the mean ± standard deviation (SD) for continuous variables and frequency (percentage) for categorical variables. Student’s *t*-test or Mann-Whitney *U* test were used to compare continuous variables, while the chi-square test or Fisher’s exact test was used to compare categorical variables between two groups. One-way analysis of variance or Kruskal-Wallis test were used for multiple group comparisons. Kaplan-Meier survival curve analysis was used for survival analysis, and a log-rank method was used to compare survival time between groups. All the statistical tests were two-sided, and a *P*-value < 0.05 was considered statistically significant. All the analyses were conducted using SPSS V22.0 and GraphPad Prism 6 software.

## Results

### POSTN is closely correlated with cancer stem cell-related molecules in HCC

**Fig. 1:**
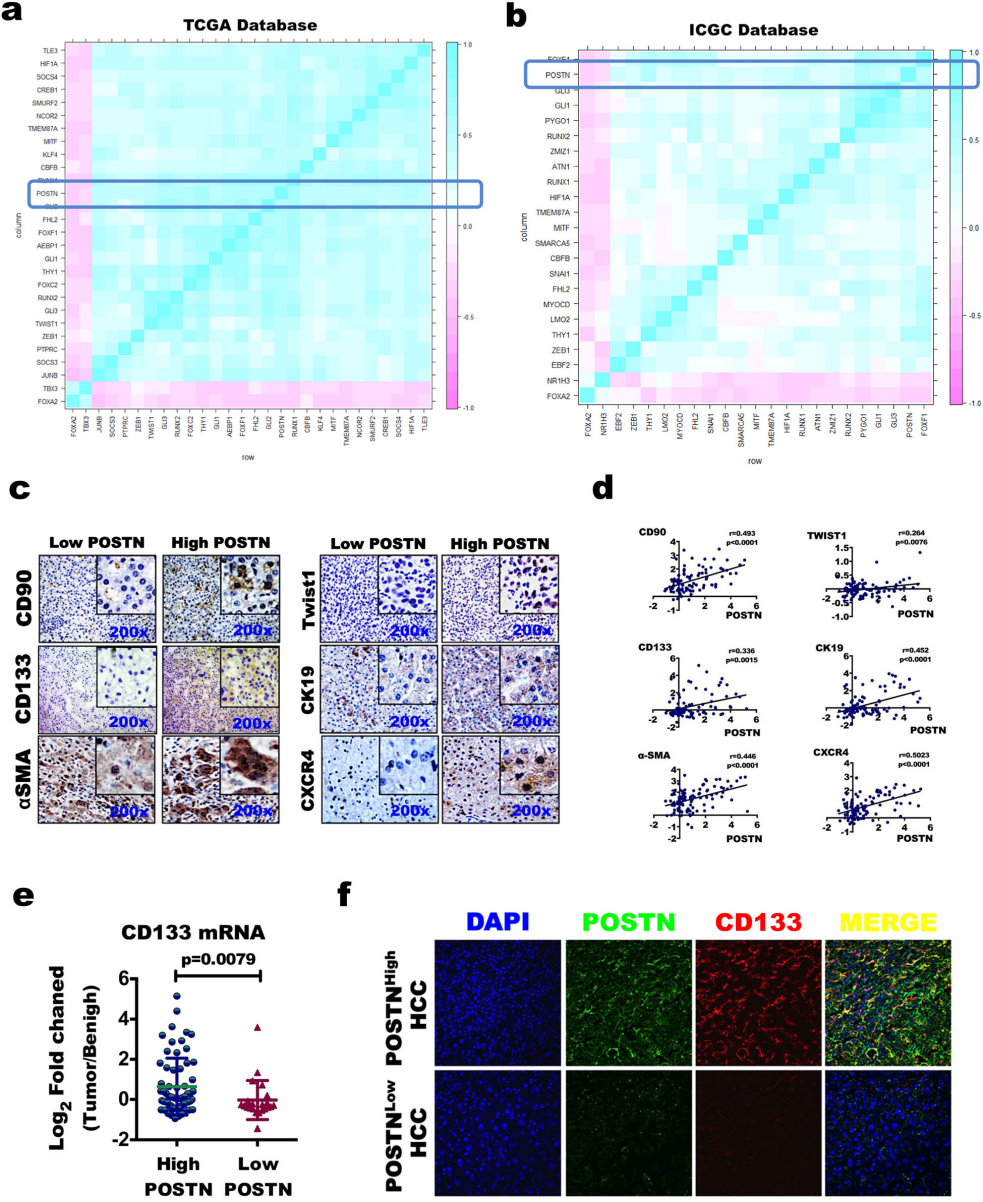
POSTN is correlated to molecular phenotype of liver cancer stem cell. **a-b** POSTN mRNA expression was positively correlated with the most common transcriptional regulators in liver cancer stem cells such as ZEB1, TWIST1, GLI1, GLI2, GLI3, RUNX1 (**a** TCGA database, **b** ICGC database). **c** Positive expression of POSTN protein in liver cancer tumor tissues were consistent with the expression of stem cell marker CD133, CD90, CK19, TWIST, CXCR4 and αSMA. **d** The expression of POSTN mRNA was also positively correlated with these six genes by using GSE1898 subset. **e** The expression of CD133 mRNA in POSTN^High^ HCC tissues was significantly higher than that in POSTN^Low^ HCC tissues. **f** POSTN (Green) could be co-expressed with CD133 (Red) in liver cancer tissues of POSTN-highly-expressing HCC by using immunofluorescence staining, CD133 was negative expression in POSTN-lowly-expressing HCC tissue. Scale bar, 50 μm

The TCGA and ICGC databases were explored to investigate the involvement of the POSTN protein in the stemness formation and maintenance of HCC cancer cells. Overall, POSTN expression was significantly positively correlated with the expression of the most common transcriptional regulatory factors in HCC cells, including *ZEB1*, *TWIST1*, *GLI1*, *GLI2*, *GLI3*, and *RUNX1* (Fig. 1A-B). IHC staining of our HCC clinical samples showed that POSTN expression changed in a direction that was consistent with the changes in the expression of other relevant proteins, including CD133, CD90, CK19, TWIST, CXCR4, and αSMA, in HCC tissues (Fig. 1C). Further analysis of the GSE1898 dataset revealed that POSTN expression was positively correlated with the expression of the six genes mentioned above (Fig. 1D); in particular, the mRNA expression level of CD133 was remarkably higher in liver cancer tissues with high POSTN expression than in liver cancer tissues with low POSTN expression (Fig. 1E). As shown in Fig. 1F, immunofluorescence staining of tissue samples obtained from cancer patients confirmed that POSTN (green) was co-expressed with CD133 (red) in liver cancer tissues with high POSTN expression. Furthermore, we found that POSTN mRNA expression was increased in 66.7% (16/24) of HCC tissues by qPCR (Fig. S1A). CD133 mRNA expression was increased in 62.5% (10/16) of POSTN-positive HCC tissues and was significantly higher than that in 12.5% (1/8) of POSTN-negative HCC tissues (Fig. S1B-C). These results indicate that the expression of POSTN is correlated with the stemness phenotype of HCC.

### The *POSTN* gene is an important factor in the regulation of the malignant behaviour and stemness transformation of HCC cells

**Fig. 2:**
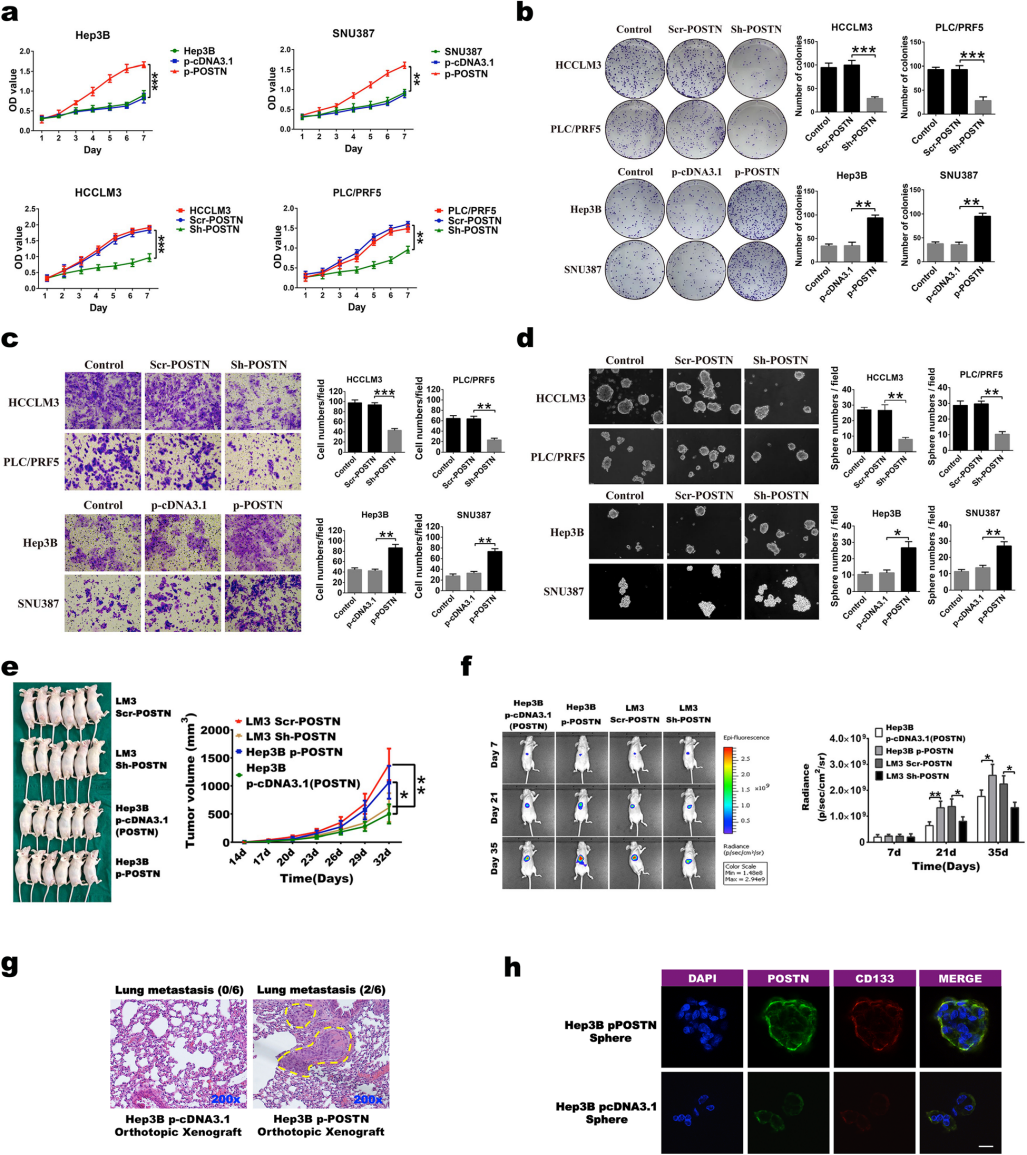
POSTN gene is correlated with the stemness transformation of liver cancer cells. **a-d** Viability (**a**), clone formation (**b**), invasion (**c**) and sphere formation (**d**) abilities were measured in POSTN^Low^ Hep3B and SNU387 cells transfected with POSTN plasmid or POSTN^High^ HCCLM3 and PLC/PRF5 cells transfected with lentiviral mediated shRNA targeting POSTN. **e** Representative photograph of the xenograft of Hep3B cells transfected with control and p-POSTN plasmid or HCCLM3 cells transfected with lentiviral packaged shRNA targeting POS TN with matched scrRNA(Left panel). Comparison of tumor growth between xenograft groups with different POSTN-expressing liver cancer cells.(Right panel). **f** Luciferase signal intensities of the mice in each group after intrahepatic injection at the indicated time points (left panel). Bar graph indicated the quantitative results of luciferase signal intensities at the indicated time points. **g** Lung metastasis were found in POSTN^High^ HCC orthotopic xenograft (Right panel). **h** Immunofluorescence dual staining was used to verify the expression of POSTN (Green) and CD133 (Red) proteins in the process of sphere formation. Scale bar, 10 μm. Data were represented as mean + SEM from three independent experiments. *P < 0.05; **P < 0.01; ***P < 0.001

Next, we measured the expression levels of the POSTN protein and various stemness-related factors in nine HCC cell lines to determine the role of upregulated POSTN expression in the stemness transformation and malignant biological behaviour of HCC cells. We found that the protein expression level of POSTN was high in the LM3, SK-Hep1, PLC/PRF5, HepG2, and HUH-7 cells but low in the Hep3B, Li-7, SNU-182, and SNU-387 cells (Fig. S2A). Therefore, in this study, the LM3 and PLC/PRF5 cell lines were used as representative cell lines with high POSTN expression, and the Hep3B and SNU-387 cell lines were used as representative cell lines with low POSTN expression. The transfection experiment using lentiviral vectors carrying *POSTN* shRNA and *POSTN* scrRNA confirmed that *POSTN* expression was successfully manipulated at the genetic level in HCC cells (Fig. S2B). Furthermore, suppressing *POSTN* gene expression could significantly inhibit the expression of stemness factors in HCC cell lines with high POSTN expression. In contrast, the upregulation of POSTN expression significantly promoted the expression of these factors in cells with low POSTN expression (Fig. S2C). At the cellular level, cell viability (Fig. 2A), colony formation (Fig. 2B), invasion (Fig. 2C), and sphere formation (Fig. 2D) were markedly reduced by inhibiting POSTN expression in cells with high POSTN expression but promoted by upregulating POSTN in cells with low POSTN expression. The in vivo experiments in subcutaneous and orthotopic transplantation mouse models demonstrated that tumour growth was suppressed by POSTN downregulation in HCC cells with high POSTN expression and enhanced by POSTN upregulation in HCC cells with low POSTN expression (Fig. 2E-F), indicating that POSTN expression is closely associated with the tumorigenicity of HCC cells. Moreover, we found pulmonary metastases in the mice implanted with cancer cells with high POSTN expression, even though there were only two mice, and no metastasis was found in the mice implanted with cancer cells with low POSTN expression (Fig. 2G). We examined POSTN (green) and CD133 (red) expression in the formed spheres using double immunofluorescence staining, and the results showed that the upregulation of POSTN expression was accompanied by the increased expression of CD133 (Fig. 2H). These results suggested that cancer cells with high POSTN expression may have an LCSC phenotype.

### AP-2α is a crucial transcription factor that mediates the effect of the POSTN protein in promoting the transformation of HCC cells into CD133^+^ LCSCs

**Fig. 3:**
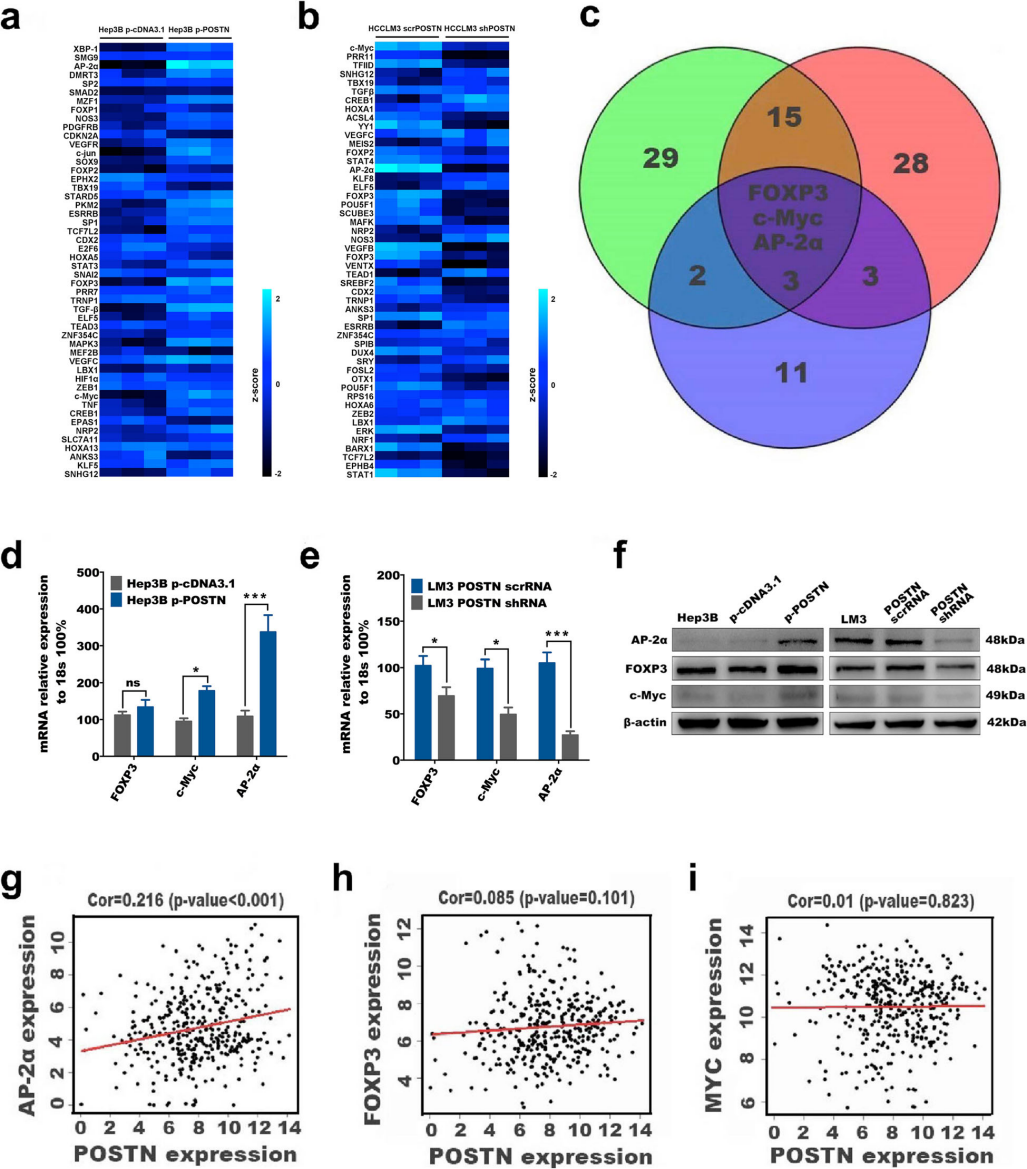
AP-2α is a key transcription factor in POSTN mediated liver cancer cell CD133-positive transformation. **a** Oligonucleotide microarray analysis results of top 50 up-regulated genes in hep3B cell after transfected with control and p-POSTN plasmid; **b** Oligonucleotide microarray analysis results of top 50 down-regulated genes in HCCLM3 cell after transfected with lentiviral packaged shRNA targeting POSTN and matched scrambled RNA; **c** Venn diagram shows genes associated with (**a**), (**b**) combined with 19 transcriptional regulators of CD133 genes predicted by the TRANSFAC transcription factor prediction database. **d-f** FOXP3, c-Myc, AP-2α mRNA (**d-e**) and protein(**f**) level were compared in Hep3B cells transfected with control or p-POSTN plasmid as well as in HCCLM3 cells transfected with lentiviral packaged shRNA targeting POSTN or scrambled RNA. Data represent mean + SEM of three independent experiments. *P < 0.05; **P < 0.01; ***P < 0.001. **g** The expression of POSTN mRNA was positively correlated with AP-2α mRNA using data from TCGA; **h-i** The expression of POSTN mRNA was negatively correlated with FOXP3 and c-Myc mRNA

To further explore the molecular mechanisms by which the POSTN protein promotes the transformation of HCC cells into CD133^+^ LCSCs, gene sequencing was used to identify the top 50 genes whose expression was altered by the upregulation of *POSTN* gene expression (Fig. 3A) and the top 50 genes whose expression was altered by downregulation of *POSTN* gene expression (Fig. 3B) in HCC cells. Among 19 transcription factors that regulate *CD133* gene expression and were identified via a TRANSFAC database-based prediction, three candidates, FOXP3, c-Myc, and AP-2α, were identified via a Venn diagram approach; that is, these three candidates were found at the intersection of all the sets in the Venn diagram and might mediate the effect of the POSTN protein in promoting HCC cells to acquire the CD133 phenotype (Fig. 3C). The qPCR and WB analysis of the mRNA and protein expression of these three transcription factors revealed that AP-2α was the most critical transcription factor involved in the POSTN-mediated regulation of CD133 expression in the HCC cell lines at the cellular level (Fig. 3D-F). Furthermore, TCGA data analysis demonstrated that POSTN expression was significantly positively correlated with AP-2α expression (Fig. 3G) but uncorrelated with FOXP3 and c-Myc expression in HCC tissues (Fig. 3H-I). This finding suggested that AP-2α plays an important role in the effect of the POSTN gene in promoting the stemness transformation and maintenance of HCC cells.

### Inhibition of AP-2α expression effectively suppresses the POSTN-induced transformation and malignant phenotype of CD133^+^ HCC cells

**Fig. 4:**
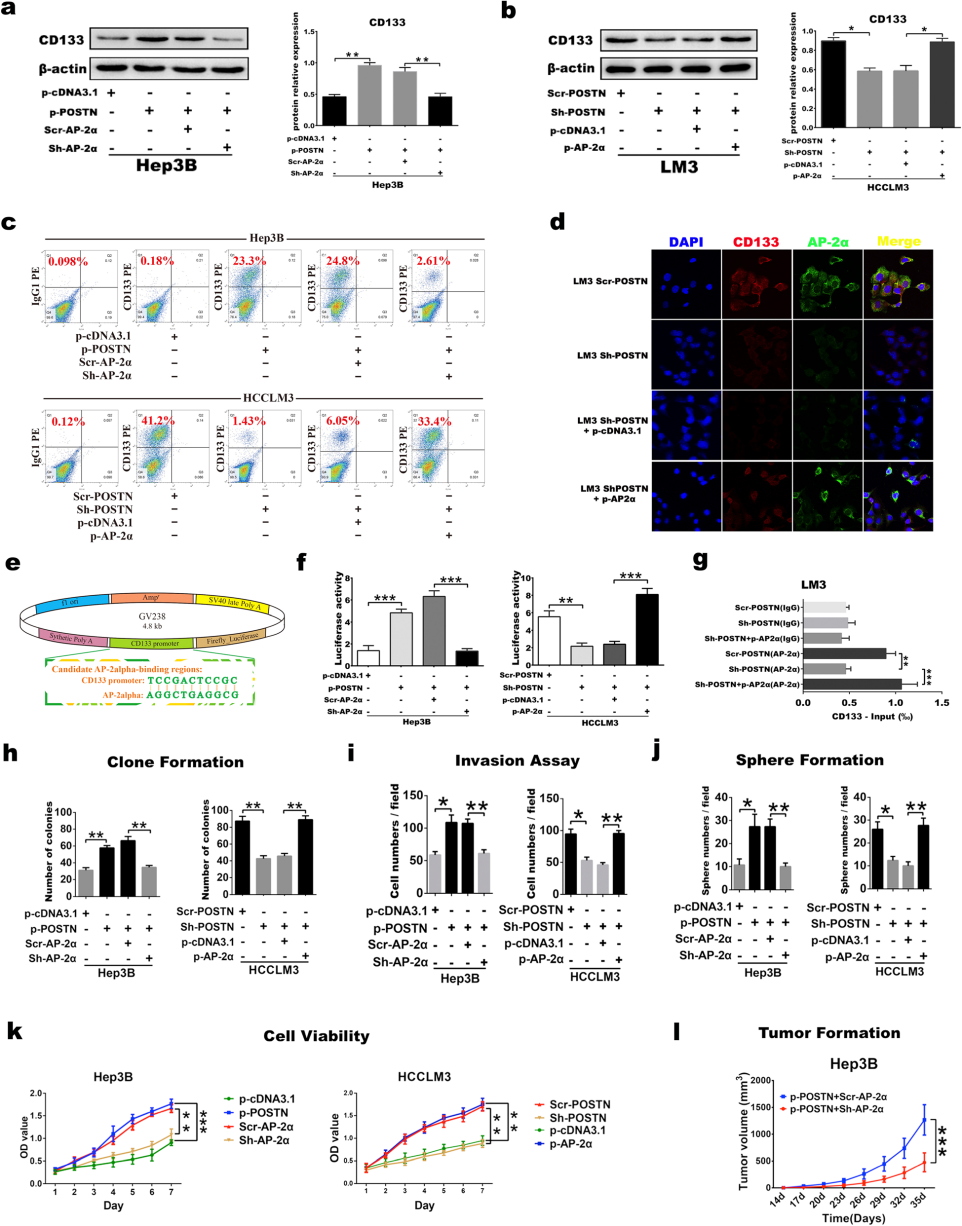
Inhibition of AP-2α expression will inhibit the transformation of CD133-positive HCC cells induced by POSTN; **a** Western Blot was used to test CD133, AP-2α, and POSTN expression in Hep3B cell after transfected with control or p-POSTN plasmid, followed by AP-2α shRNA to target AP-2α. **b** CD133, AP-2α, and POSTN protein expression was carried out by rescue experiment targeting POSTN and then recovered AP-2α expression in POSTN-expressing LM3 cell. **c** Flow Cytometry Analysis was used to quantify CD133-positive hepatoma cells number after transfected with control or p-POSTN plasmid, followed by AP-2α shRNA to target AP-2α as well as targeting POSTN and then recovered AP-2α expression in POSTN-expressing HCCLM3 cell. **d** Immunofluorescence dual staining was used to verifiy that after down-regulation of POSTN expression in hepatoma cells, restoring AP-2α (Green) expression was still effective in up-regulating CD133 (Red) expression induced by POSTN. Scale bar, 10 μm. **e** The reporter plasmid map of double luciferase contains the promoter region sequence of CD133 gene, which can be matched with AP-2α partial base complementary. **f** The Luciferase Report experiment confirmed that inhibition of AP-2α expression would effectively inhibit the luciferase activity of CD133 promoter caused by POSTN, and recovery of AP-2α expression in the POSTN down-regulated HCC cells would promote the luciferase activity of CD133 promoter. **g** ChIP experiment further confirmed the direct binding of AP-2α to CD133 promoter region in LM3 cells, IgG and AP-2α in parenthesis stated the targeting of antibody used in chromatin immunoprecipitation assay; **h-k** Clone formation (**h**), Invasion (**i**), Sphere formation ability (**j**) and viability (**k**) of hepatoma cells were measured after transfected with control or p-POSTN plasmid, followed by AP-2α shRNA to target AP-2α as well as targeting POSTN and then recovered AP-2α expression in POSTN-expressing LM3 cell. **l** Inhibition of AP-2α expression will effectively inhibit the high tumorigenicity of POSTN in HCC cells。Data were represented as mean + SEM from three independent experiments. *P < 0.05; **P < 0.01; ***P < 0.001

To explore the role of the transcription factor AP-2α in regulating the POSTN-induced stemness transformation and maintenance of HCC cells, AP-2α expression was manipulated by using lentiviral vectors carrying AP-2α shRNA or a AP-2α-encoding gene. Hep3B cells with low POSTN expression were co-transfected with plasmids carrying or not carrying the POSTN-encoding gene (p-POSTN or p-cDNA3.1) and AP-2α-scrRNA or AP-2α-shRNA lentiviruses. Similarly, LM3 cells with high POSTN expression were co-transfected with POSTN-shRNA or scrRNA lentiviruses and plasmids carrying or not carrying the AP-2α-encoding gene (p-AP-2α or p-cDNA3.1). The WB results confirmed that the POSTN-enhanced CD133 expression was downregulated by the inhibition of AP-2α expression in HCC cells (Fig. 4A), and CD133 expression could be rescued by restoring AP-2α expression in HCC cells with downregulated POSTN expression (Fig. 4B). Flow cytometry showed that the inhibition of AP-2α expression effectively inhibited the POSTN-induced increase in the numbers of CD133^+^ cells, while AP-2α overexpression increased the numbers of CD133^+^ HCC cells (Fig. 4C). Double immunofluorescence staining showed that enhancing AP-2α expression in HCC cells by downregulating POSTN expression could effectively increase the POSTN-induced expression of CD133 (Fig. 4D).

To further investigate whether the POSTN-enhanced expression of AP-2α directly transcriptionally regulates CD133 expression, dual-luciferase reporter plasmids containing the CD133 promoter sequence (Fig. 4E) were transfected into HCC cells with different POSTN and AP-2α expression levels. The results showed that the inhibition of AP-2α expression effectively suppressed the POSTN-induced luciferase activity of the CD133 promoter, and the restoration of AP-2α expression enhanced the CD133 promoter reporter-derived luciferase activity in HCC cells with decreased POSTN expression (Fig. 4F). ChIP assays confirmed that AP-2α can directly bind to the CD133 promoter (Fig. 4G).

Next, the role of AP-2α in the POSTN-induced stemness transformation and malignant behaviours of HCC cells was explored to determine the feasibility of AP-2α-based targeted therapy in the treatment of HCC. The results showed that the malignant biological behaviours of HCC cells, including proliferation, invasion, colony formation and sphere formation, were enhanced by upregulating AP-2α gene expression in HCC cells with low POSTN expression and suppressed by downregulating AP-2α gene expression (Fig. 4I-K). In addition, AP-2α inhibition effectively inhibited the tumorigenicity of HCC cells in vivo (Fig. 4L).

### POSTN promotes the release of TGFβ1 and then activate its own expression and secretion by activating αvβ3

**Fig. 5:**
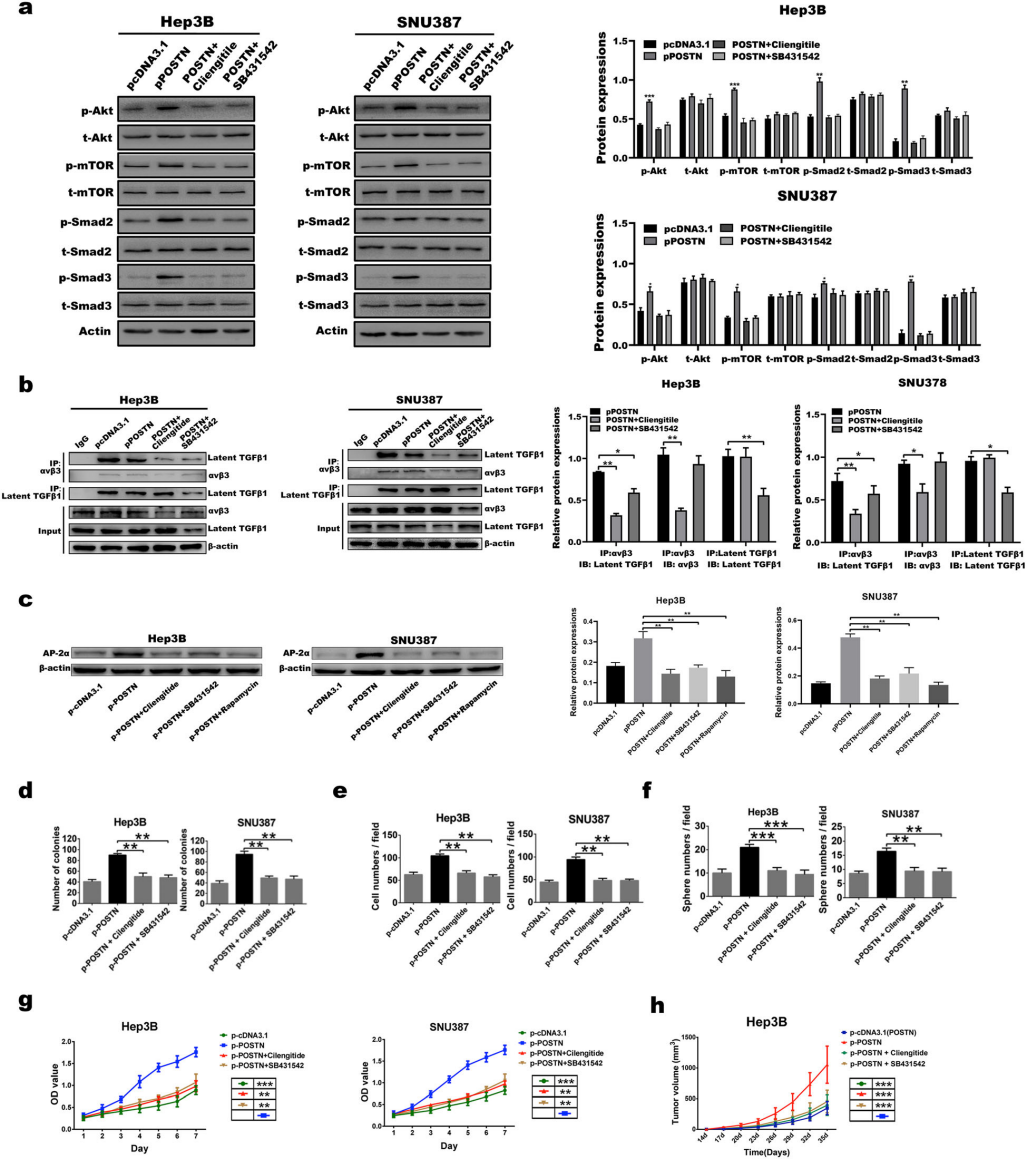
POSTN protein can release TGFβ1 by activating αvβ3, thereby activating its own expression and secretion; **a** WB and its corresponding grayscale calculation were used to identify the protein expression in Hep3B and snu387 cells after up-regulate POSTN expression, followed by using cilengitide to target αvβ3 and SB431542 to inhibit TGFβ1 receptor. **b** CO-IP pull down experiment showed αvβ3/LTAB complex in Hep3B and snu387 cells as well as up-regulate POSTN expression, followed by using cilengitide to target αvβ3 and SB431542 to inhibit TGFβ1. **c** AP-2α protein expression was examined by WB experiment after up-regulate POSTN expression, followed by using cilengitide to target αvβ3, SB431542 to inhibit TGFβ1, and rapamycin to inhibit mTOR, ***P < 0.001; **d-g** Clone Formation (**d**), Invasion (**e**), Sphere Formation ability (**f**), and viability (**g**) of hepatoma cells were measured after up-regulate POSTN expression, followed by using cilengitide to target αvβ3 and SB431542 to inhibit TGFβ1. Data represent mean + SEM of three independent experiments, the significance between groups were labeled beside the group mark. *P < 0.05, **P < 0.01, ***P < 0.001; **h** In vivo transplanted xenograft verified that blocking the positive feedback loop formed by POSTN/TGFβ1 with Cilengitide or SB431542 would effectively inhibit the high tumorigenic ability of POSTN in HCC cells, the significance between groups were labeled beside the group mark, ***P < 0.001

Our previous research showed that TGFβ1 can promote POSTN expression and secretion by prompting its downstream gene, *Smad3,* to directly bind to the POSTN promoter region [13]. Here, we further investigated whether the POSTN protein can in turn upregulate TGFβ1 expression and secretion and whether a POSTN/TGFβ1 positive feedback loop can be formed to promote the stemness transformation and maintenance of HCC cells. First, the overexpression of POSTN using p-POSTN-encoding plasmids increased the levels of phosphorylated TGFβ1 and its downstream proteins Smad-2, Smad-3, and mTOR in both Hep3B and SNU387 cells, and the POSTN-induced activation of signals downstream of TGFβ1 was inhibited by the αvβ3 antagonist cilengitide and the TGFβ1 receptor inhibitor SB431542 (Fig. 5A). Under normal conditions, TGFβ1 is stored in the extracellular matrix in a form of inactive complexes formed by latent TGF-β-binding protein (LTAB) and αvβ3. The activation of αvβ3 liberates active TGFβ1 from the complexes, leading to the activation of its downstream signalling pathway [21]. In this study, Co-IP assay revealed that the overexpression of POSTN did not affect the expression of avβ3 or LTAB, but the level of αvβ3/LTAB complexes, which was high on the membranes of control Hep3B cells with low POSTN expression, could be significantly reduced by the POSTN-mediated activation of the αvβ3 pathway in POSTN-overexpressing Hep3B cells. This decrease could be intensified by the application of cilengitide and SB431542 (Fig. 5B). Consistent with the results in Fig. 3F, targeting POSTN activation with the three inhibitors markedly suppressed the POSTN-induced AP-2α expression (Fig. 5C), and targeting POSTN also suppressed the POSTN-induced CD133 expression (Fig. S3A-B). Both in vitro and in vivo experiments further confirmed that blocking the αvβ3/TGFβ1 pathway could inhibit the proliferation, invasion, colony formation, sphere formation and tumorigenesis of HCC cells induced by the upregulation of POSTN (Fig. 5D-H). Taken together, POSTN can activate the αvβ3 receptor and liberate active TGFβ1 from the αvβ3/LTAB complex, and then, TGFβ1 induces the direct binding of its downstream smad3, to the POSTN promoter to increase the expression and secretion of POSTN. Through this positive feedback loop, POSTN plays an important role in promoting the stemness transformation and maintenance of HCC cells.

### Targeting the POSTN/αvβ3 pathway effectively inhibits tumour growth in the in vivo PDX model with high POSTN expression

**Fig. 6:**
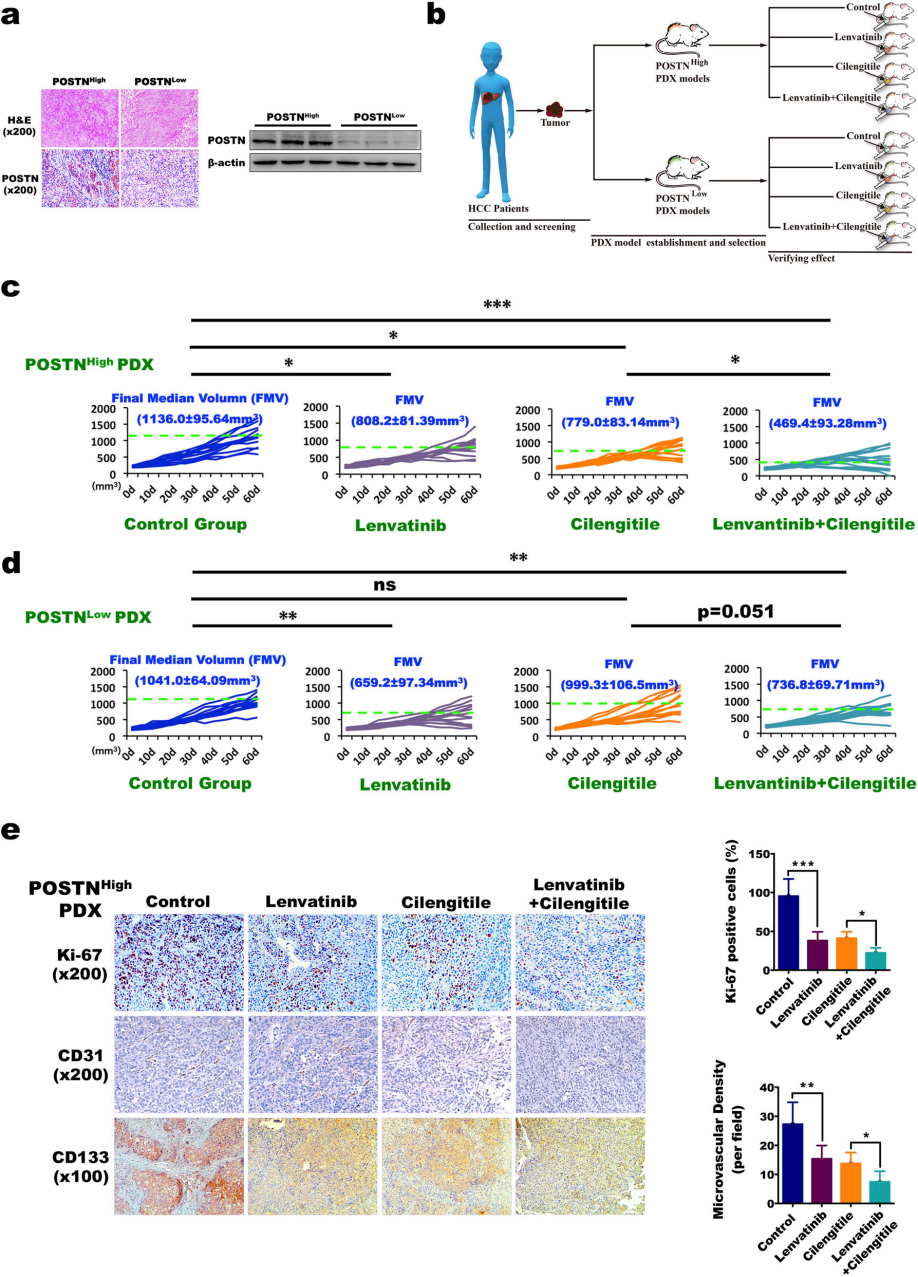
In the POSTN^High^ PDX liver cancer model, targeting POSTN/αVβ3 pathway can effectively inhibit tumor growth. **a** Experimental diagram; **b** POSTN immunohistochemical staining (Left panel) and WB (Right panel) confirmed the successful construction of POSTN High and POSTN Low PDX tumor models; **c** In POSTN^High^ PDX model, the inhibitory effects of cilengitide and lenvatinib on the growth of liver cancer were detected; FMV represents Final Medium Volume; *n* = 12, *P < 0.05, ***P < 0.001; **d** In POSTN^Low^ PDX model, the inhibitory effects of cilengitide and lenvatinib on the growth of liver cancer were detected; FMV represents Final Medium Volume; *n* = 12, **P < 0.01, ns means no significant; **e** The expression of Ki-67, CD31 and CD133 were detected by immunohistochemistry. The number of Ki-67 positive cells and microvascular density (MVD) were detected by quantitative analysis on the right bar graph, Data represent mean + SEM of three independent experiments. *P < 0.05, **P < 0.01, ***P < 0.001

Cilengitide, an FDA-approved αvβ3 pathway-specific antagonist, and lenvatinib, currently the most commonly used first-line drug in the targeted therapy of advanced HCC, were used to explore the effect of inhibiting the POSTN/αvβ3 pathway on the growth of HCC. The PDX models with different POSTN expression levels, i.e., POSTN^High^ PDX and POSTN^Low^ PDX, were established, and their POSTN protein expression was verified by IHC staining and WB assay (Fig. 6A). All the POSTN^High^ PDX and POSTN^Low^ PDX mice were randomly divided into four groups that received different treatments when the xenografts reached volumes of approximately 200 mm^3^; these groups included the control group, the lenvatinib group, the cilengitide group, and the lenvatinib + cilengitide group (Fig. 6B). After weekly intragastric administration and intraperitoneal injection for 60 days, the final median volume of the tumours in the POSTN^High^ mice was significantly decreased by lenvatinib (808.2 ± 81.39 mm^3^) and cilengitide (779.0 ± 83.14 mm^3^) compared with the control (1136.0 ± 95.64 mm^3^); in particular, the tumour volume was decreased most notably by the combination of the two drugs (469.4 ± 93.28 mm^3^), showing a statistically significant difference when compared with either drug alone and the control (Fig. 6C). However, in the POSTN^Low^ mice, lenvatinib only demonstrated a weak therapeutic effect (659.2 ± 97.34 mm^3^), while neither cilengitide (999.3 ± 106.5 mm^3^) nor the combined use of the two drugs (736.8 ± 69.71 mm^3^) resulted in any statistically significant difference compared with the control (1041.0 ± 64.09 mm^3^) (Fig. 6D). IHC staining of the tumour tissues harvested from the POSTN^High^ mice showed that the proteins expression levels of Ki67 and CD31 were markedly reduced by lenvatinib and cilengitide (Fig. 6E left panel). The number of Ki67-positive cells and the microvascular density were both most significantly decreased by the combined use of lenvatinib and cilengitide (Fig. 6E right panel), suggesting a strong inhibitory effect on cancer cell proliferation and angiogenesis in HCC. The results proved that inhibiting the activity of the POSTN/αvβ3 pathway in HCC cells with high POSTN expression can effectively inhibit tumour growth, and the combined use of cilengitide and lenvatinib has a higher treatment efficacy.

### Co-expression of POSTN and AP-2α is an important indicator of poor prognosis in HCC patients

**Fig. 7:**
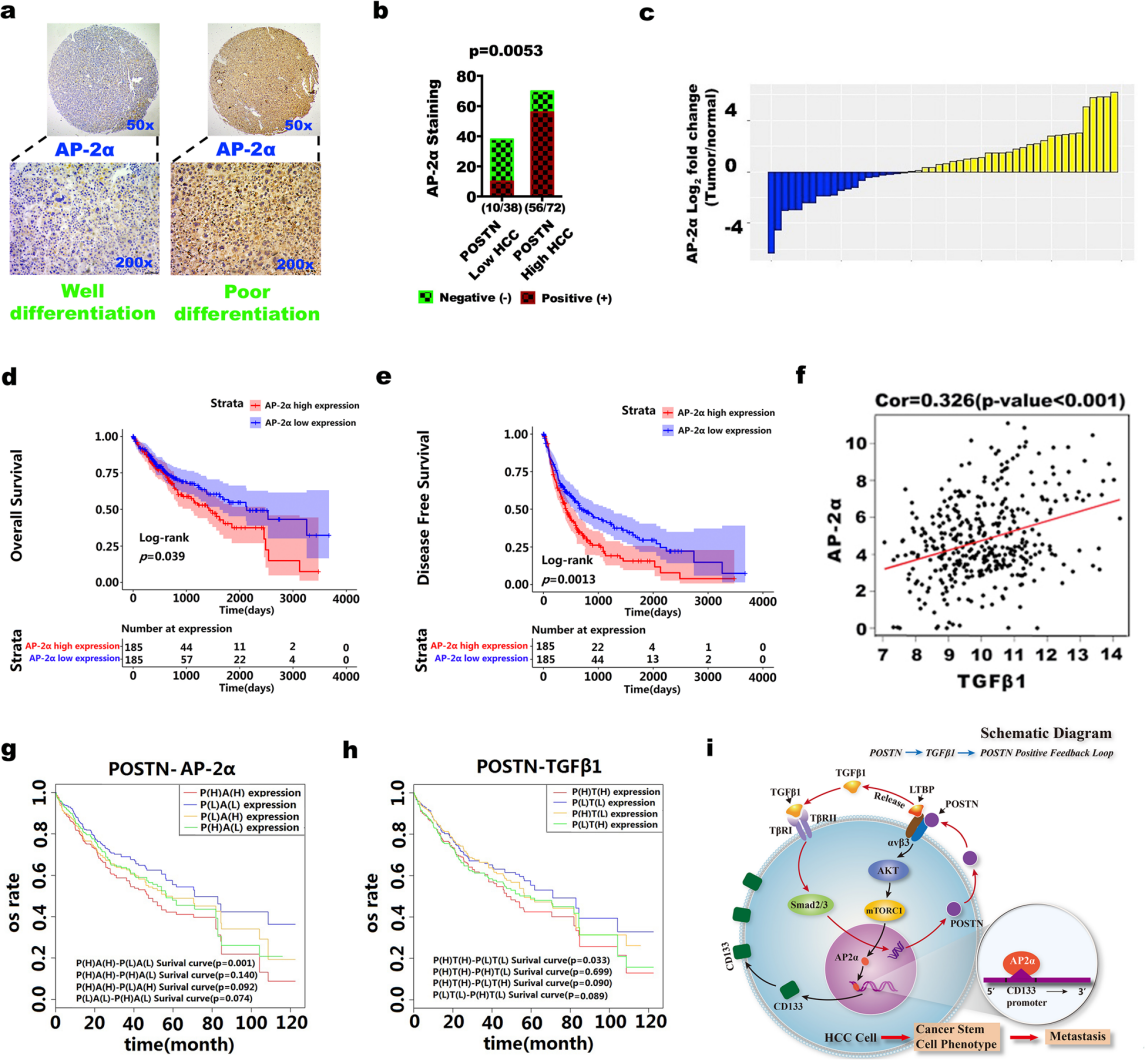
Co-expression of POSTN and AP-2α in liver cancer is an important indicator of poor prognosis. **a** Immunohistochemical staining was used to detect the expression of AP-2α in liver cancer tissue chip. The left side was highly differentiated liver cancer tissue, and the right side was poorly differentiated liver cancer tissue. The magnification of the image above was 50x, and the image below was 200x; **b** The staining of AP-2α protein in POSTN^High^ and POSTN^Low^ HCC tissues were compared by Chi square test; **c** Waterfall plot demonstrated that AP-2α overexpression were found in 68% of HCC cases; **d** Analysis of TCGA data revealed that HCC patients with high AP-2α expression (*Z* score > 1) were significantly associated with poorer overall survival; **e** Analysis of TCGA data revealed that HCC patients with high AP-2α expression (*Z* score > 1) were significantly associated with poorer disease-free survival; **f** Analysis of TCGA data revealed that the expression of AP-2α mRNA was positively correlated with the expression of TGFβ1 mRNA; **g** Using POSTN and AP-2α stratification analysis to confirm that HCC patients with high expression levels of both periostin and AP-2α had the worst prognosis; **h** Using POSTN and TGFβ1 stratification analysis to confirm that HCC patients with high expression levels of both POSTN and TGFβ1 had the worst prognosis compared to that of the Lower periostin/TGFβ1 group; **i** The flow chart of POSTN/TGFβ1 positive feedback pathway using AP-2α to promote the transformation and malignant process of liver cancer cells

Using tissue microarray (TMA) technology, POSTN and AP-2α expression profiling in HCC tissue was performed by double staining to explore the possible roles of POSTN and AP-2α in the early diagnosis and prognosis of HCC patients. All the samples were obtained from 110 patients who underwent surgical resection of primary tumours in the Department of Hepatobiliary Surgery, The First Affiliated Hospital of Wenzhou Medical University, between November 2008 and October 2010. The results showed that among the 110 HCC samples, 72 (65.5%) were POSTN-positive and 66 (60.0%) were AP-2α-positive. In addition, AP-2α expression was significantly higher in poorly differentiated HCC than in highly differentiated HCC, and AP-2α expression was positive in a significantly higher percentage of patients with high POSTN expression (56/72, 77.8%) than in patients with low POSTN expression (10/38, 26.3%) (Fig. 7A-B). According to the data extracted from the TCGA database, AP-2α expression was significantly higher in the tumour tissues than in the corresponding adjacent tissues in 68.3% of patients (Fig. 7C). Survival analysis using overall HCC data from TCGA database showed that the overall survival rate (Fig. 7D) and disease-free survival rate (Fig. 7E) were lower in patients with high AP-2α expression than in those with low AP-2α expression. In addition, TCGA data revealed a positive correlation between the AP-2α and TGFβ1 expression levels in HCC patients (Fig. 7F). The patient cohort in this study was further categorized into four groups based on the expression levels of POSTN and AP-2α, i.e., the high-POSTN and high-AP-2α group, the high-POSTN and low-AP-2α group, the low-POSTN and low-AP-2α group, and the low-POSTN and high-AP-2α group. The results revealed that HCC patients with high expression of both POSTN and AP-2α had a median survival time (MS) of 21.1 months after tumour resection, which was the worst survival time observed in all the groups and was significantly shorter than the survival time of patients with low expression of both AP-2α and POSTN (MS 43.2 months) (*P* = 0.001) (Fig. 7G). Similarly, the patient cohort was categorized into four groups based on the expression levels of POSTN and TGFβ1 for prognostic analysis. The low-POSTN and low-TGFβ1 group (MS 45.6 months) had a longer survival than the high-POSTN and low-TGFβ1 group (MS 28.9 months) (*P* = 0.033) (Fig. 7H). Figure 7I is a schematic diagram showing that the POSTN/TGFβ1 positive feedback loop promotes the stemness characterization of HCC cells via AP-2α-triggered CD133 transcription. These results suggest that the oncogenesis of both AP-2α and POSTN as well as POSTN and TGFβ1 is dependent on the presence of the other protein and support our findings that the POSTN/TGFβ1 positive feedback loop activated AP-2α expression and is a key mediator of the oncogenic effect of POSTN in HCC.

## Discussion

Tumour stem cells play important roles in the metastasis, recurrence, and chemotherapy resistance of HCC. However, the origin and mechanism of production of these cells remain unclear. Based on our previous research findings showing that the *POSTN* gene plays a vital role in the malignant progression and metastasis of HCC, this study further confirmed that *POSTN* can promote CD133^+^ LCSC production and stemness maintenance by using various bioinformatics and molecular biological tools. The transcription factor AP-2α was proven to be the key factor that mediates the POSTN-induced stemness transformation of HCC cells, while the positive feedback loop formed by POSTN and TGFβ1 was the key regulatory pathway for stemness maintenance in LCSCs.

The mechanism underlying LCSC generation has not been clearly defined. Some researchers believe that the CSCs in many tumours, including HCC, are derived from the abnormal proliferation and differentiation of normal stem cells [7]. Zeng et al. showed that CD133^+^ haematopoietic stem cells may evolve into LCSCs [22]. A number of recent studies reported that CSCs were produced by differentiated and mature tumour cells in response to various inducing factors [8–10]. CD133 is a known CSC marker. Song and Terai et al. found that CD133^+^ HCC cells had stronger invasion, metastasis and tumorigenicity abilities in vivo and proposed that CD133 might be an LCSC marker [23, 24]. The transcription factor STAT3 can directly bind to the CD133 promoter region [25]. Lin et al. and Wu et al. found that the expression of CD133 in HCC cells is regulated by the IL6/STAT3 and Hedgehog signalling pathways [26, 27] However, the specific mechanism underlying the regulation of CD133 transcription in HCC cells remains unclear. In this study, we found that the upregulated protein expression of POSTN in the tumour microenvironment promoted the direct binding of the transcription factor AP-2α to the CD133 promoter region and therefore enhanced the expression of the CD133 gene in HCC cells. This process involved a TGFβ1-mediated positive feedback signalling pathway. Targeting AP-2α effectively inhibited the POSTN-induced CD133 upregulation and stemness development in HCC cells. Previous studies have shown that AP-2α plays an important role in the formation and function of stem cells [28]. AP-2α is a major contributor involved in tumour resistance to chemotherapy [29]. However, there are discrepant findings regarding the role of AP-2α in tumour occurrence and development. AP-2α accelerates the progression of cholangiocarcinoma [30] but inhibits the growth of colorectal cancer [31], and both promoting and inhibitory effects on HCC have been reported [32, 33]. In this study, we analysed tissue samples from 110 HCC patients and found that AP-2α expression was significantly upregulated in 60% of the patients, which was consistent with the findings in TCGA database. Survival analysis confirmed that the expression of AP-2α was significantly positively correlated with the prognosis of these patients. Akt and focal adhesion kinase (FAK) are important signalling pathways downstream of POSTN [34, 35]. AMP-activated protein kinase (AMPK), a kinase of Akt, can activate AP-2α [36]. Therefore, it is reasonable to conclude that the POSTN protein can increase AP-2α phosphorylation via the Akt/AMPK pathway.

Positive feedback activation is an important mechanism underlying the maintenance of stem cell stemness [37, 38]. The POSTN levels in the tumour microenvironment are significantly increased in various tumours [14], and POSTN plays an important role in the stemness maintenance of stem cells [39]. This study explored whether a positive feedback pathway contributes to high POSTN expression in the HCC microenvironment and the stemness maintenance of LCSCs. Our previous research revealed that Sulf2 can stimulate the expression and secretion of POSTN through TGFβ1 in the HCC tumour microenvironment [13]. TGFβ1 is an important regulatory factor in the formation of stem cells [40, 41]. This implies that the interaction of POSTN and TGFβ1 may be critical for the formation of LCSCs in HCC. However, the question of whether high expression of POSTN can in turn activate the expression and secretion of TGFβ1 remains to be addressed. TGFβ1 is stored in the extracellular matrix in the form of LTAB by forming complexes with αvβ3. Activation of αvβ3 prompts TGFβ1 to be released into the tumour microenvironment in its active form [21]. αvβ3 is a classical receptor of the POSTN protein [34, 42]. In this study, upregulated POSTN protein expression activated, and bound to the αvβ3 receptor on the surface membranes of HCC cells, which prompted the expression and release of TGFβ1. TGFβ1 bound to its receptors on the cell surface membranes to promote the expression and secretion of the POSTN protein, forming a positive feedback loop between POSTN and TGFβ1. This positive feedback loop promoted the stemness transformation and maintenance of HCC cells. In addition, the upregulation and activation of αvβ3 is also an important factor that contributes to the generation of tumour stem cells [42, 43]. Thus, our study provided an integrated molecular pathway of LCSC formation and the maintenance of stemness features, which illuminates further investigation into the role of LCSCs in HCC.

Our study also showed the clinical significance of this basic research. By using the αvβ3 receptor inhibitor cilengitide and the TGFβ1 inhibitor SB431542 at both the cellular level and in the PDX model in vivo*,* both inhibitors presented intense effects in reversing the POSTN-induced stemness transformation and malignant progression of HCC. In particular, the PDX model was established by implanting human primary tumour cells directly into immunocompromised mice, which retains the characteristics of the patient’s tissue. This model allows a better understanding of the oncogenic pathways and cellular interactions in the heterogenetic tumour sample and is an excellent model for testing antitumour treatments in vivo [44, 45]. Cilengitide, an αvβ3 receptor inhibitor, can block anoikis resistance in HCC [46]. Currently, a clinical study on the use of cilengitide for treating recurrent HCC is underway; however, it still needs additional basic theoretical support. This study provided a theoretical basis for the application of cilengitide combined with lenvatinib, which is the first-line drug used in the targeted therapy of HCC, in the postoperative prevention and treatment of HCC with high POSTN expression. In addition, an independent phase II study with newly diagnosed patients with glioblastoma multiforme (GBM) revealed that cilengitide combined with chemotherapy and radiotherapy (e.g., temozolomide plus radiotherapy) could prolong the survival of patients [47]. This finding provides new supporting evidence and a strategy for the individualized treatment of HCC.

## Conclusions

This study concluded that high POSTN levels in the tumour microenvironment resulting from the POSTN/TGFβ1 positive feedback loop can activate AP-2α to transcriptionally induce the expression of CD133, which promotes the stemness transformation of HCC cells. The POSTN/TGFβ1/AP-2α pathway might be a promising target bringing a breakthrough for the targeted therapy of HCC.

## Supplementary Information

**Additional file 1: Fig. S1.** (**a**) The expression of POSTN mRNA in 24 cases of liver cancer and its adjacent tissues; (**b**) The expression of CD133 mRNA in 16 cases of POSTN positive liver cancer and 8 cases of POSTN negative liver cancer; (**c**) The comparison of the positive rate of CD133 mRNA in POSTN positive liver cancer and that in POSTN negative liver cancer. Data represent mean + SEM of three independent measurements of mRNA levels carried out the same liver specimen. *P < 0.05, **P < 0.01, ***P < 0.001; ns means no significant.

**Additional file 2: Fig. S2.** (**a**) POSTN protein expressed in 9 HCC cell lines, β-actin as inner control; (**b**) The transfection experiment confirmed that POSTN expression was manipulated successfully at a genetic level in HCC cells, Data represent mean + SEM of three independent experiments. ***P < 0.001; (**c**) Upregulation of POSTN gene expression can promote the expression of stem cell related genes (E-cadherin, N-cadherin, vimentin, Twist1, Snail1 and α SMA) in HCC cells, downregulation of POSTN gene expression can inhibit the protein expression of these genes.

**Additional file 3: Fig. S3.** (**a-b**) CD133 protein expression was examined by WB (**a**) experiment and (**b**) Immunofluorescence staining after up-regulate POSTN expression, followed by using cilengitide to target αvβ3 and SB431542 to inhibit TGFβ1, ***P < 0.001.

**Additional file 4: Fig. S4.** Protein structure and binding site of POSTN.

**Additional file 5: Table S1.** List of antibodies, reagents and kits used in the study. **Table S2.** Primer of genes used in this article.

## Data Availability

All data and materials supporting the findings of this work are available from its supplementary information files and from the corresponding author upon reasonable request.

## Abbreviations

AMPK: AMP-activated protein kinase
AP-2α: Activator protein-2α
CSCs: Cancer stem cells
FCM: Flow cytometry
FDA: Food and drug administration
FOXP3: Forkhead box protein P3
FVM: Final median volume
HCC: Hepatocellular carcinoma
ICGC: International cancer genome consortium
IHC: Immunohistochemistry
IL6: Interleukin 6
LCSCs: Liver cancer stem cells
LTAB: Latent TGF-β binding protein
MS: Median survival
MVD: Microvascular density
PDX: Patient-derived xenograft
POSTN: Periostin
STAT3: Signal transducer and activator of transcription 3
TCGA: The cancer genome atlas
TGFβ1: Tumor necrosis factor (TGF)-β1
TMA: Tissue microarray
