# Supplementary figures and images for: A positive feedback loop between Periostin and TGFβ1 induces and maintains the stemness of hepatocellular carcinoma cells via AP-2α activation
Source: J Exp Clin Cancer Res. 2021 Jun 30;40:218. doi: 10.1186/s13046-021-02011-8 (PMC8243733; doi:10.1186/s13046-021-02011-8)

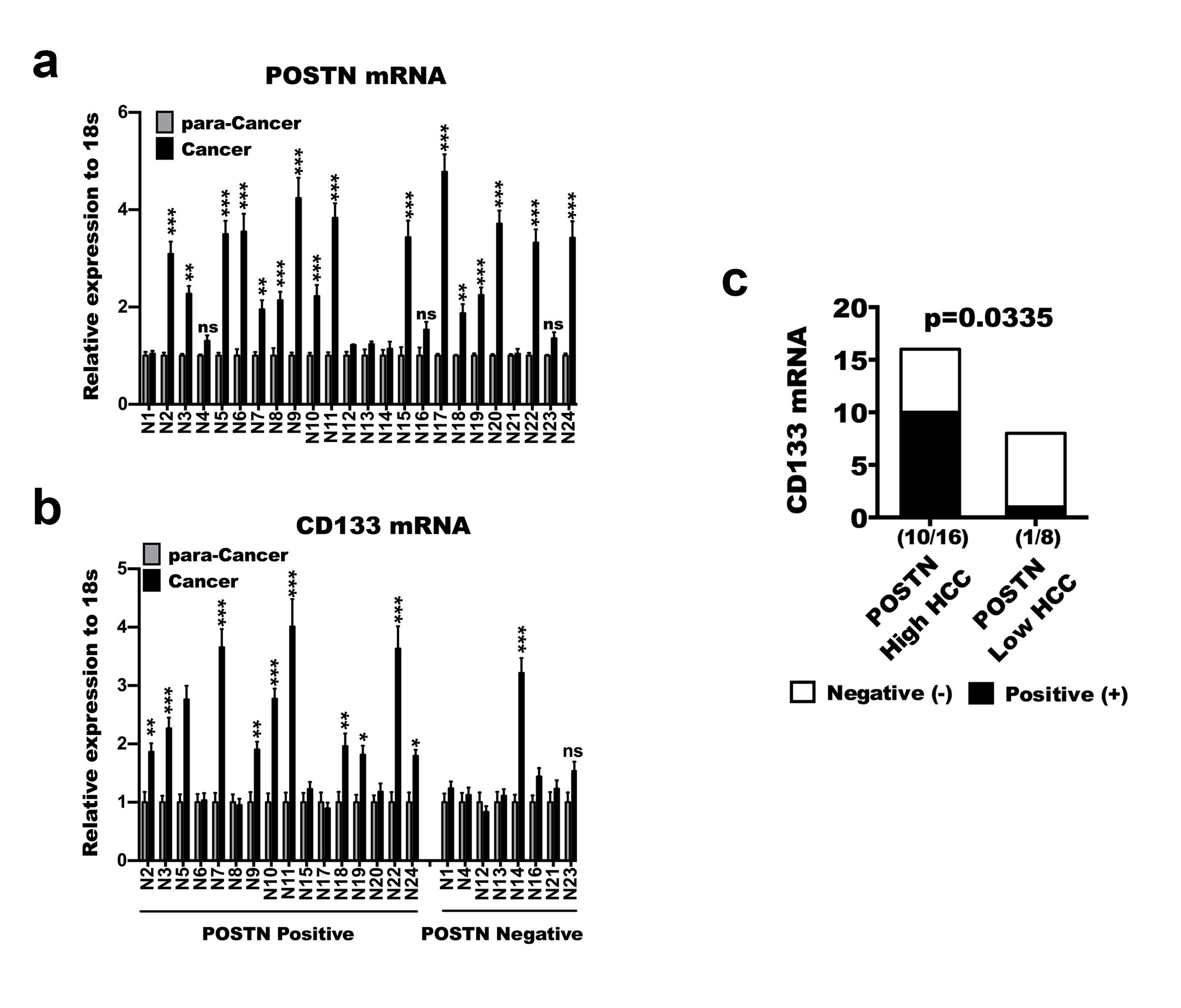

Supplement: Supplementary file 1 — Additional file 1: Fig. S1. (a) The expression of POSTN mRNA in 24 cases of liver cancer and its adjacent tissues; (b) The expression of CD133 mRNA in 16 cases of POSTN positive liver cancer and 8 cases of POSTN negative liver cancer; (c) The comparison of the positive rate of CD133 mRNA in POSTN positive liver cancer and that in POSTN negative liver cancer. Data represent mean + SEM of three independent measurements of mRNA levels carried out the same liver specimen. *P < 0.05, **P < 0.01, ***P < 0.001; ns means no significant. [file 13046_2021_2011_MOESM1_ESM.jpg]

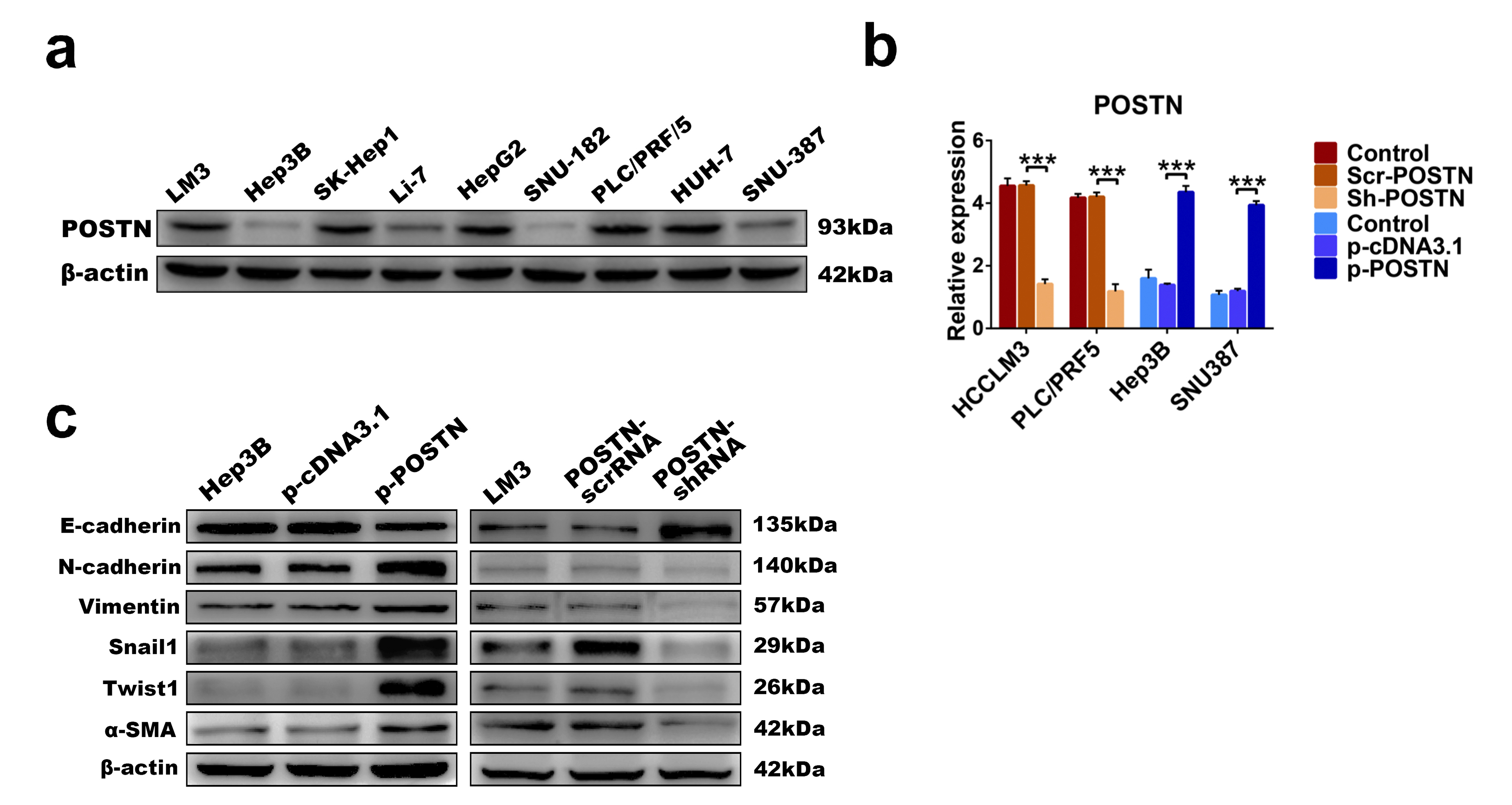

Supplement: Supplementary file 2 — Additional file 2: Fig. S2. (a) POSTN protein expressed in 9 HCC cell lines, β-actin as inner control; (b) The transfection experiment confirmed that POSTN expression was manipulated successfully at a genetic level in HCC cells, Data represent mean + SEM of three independent experiments. ***P < 0.001; (c) Upregulation of POSTN gene expression can promote the expression of stem cell related genes (E-cadherin, N-cadherin, vimentin, Twist1, Snail1 and α SMA) in HCC cells, downregulation of POSTN gene expression can inhibit the protein expression of these genes. [file 13046_2021_2011_MOESM2_ESM.jpg]

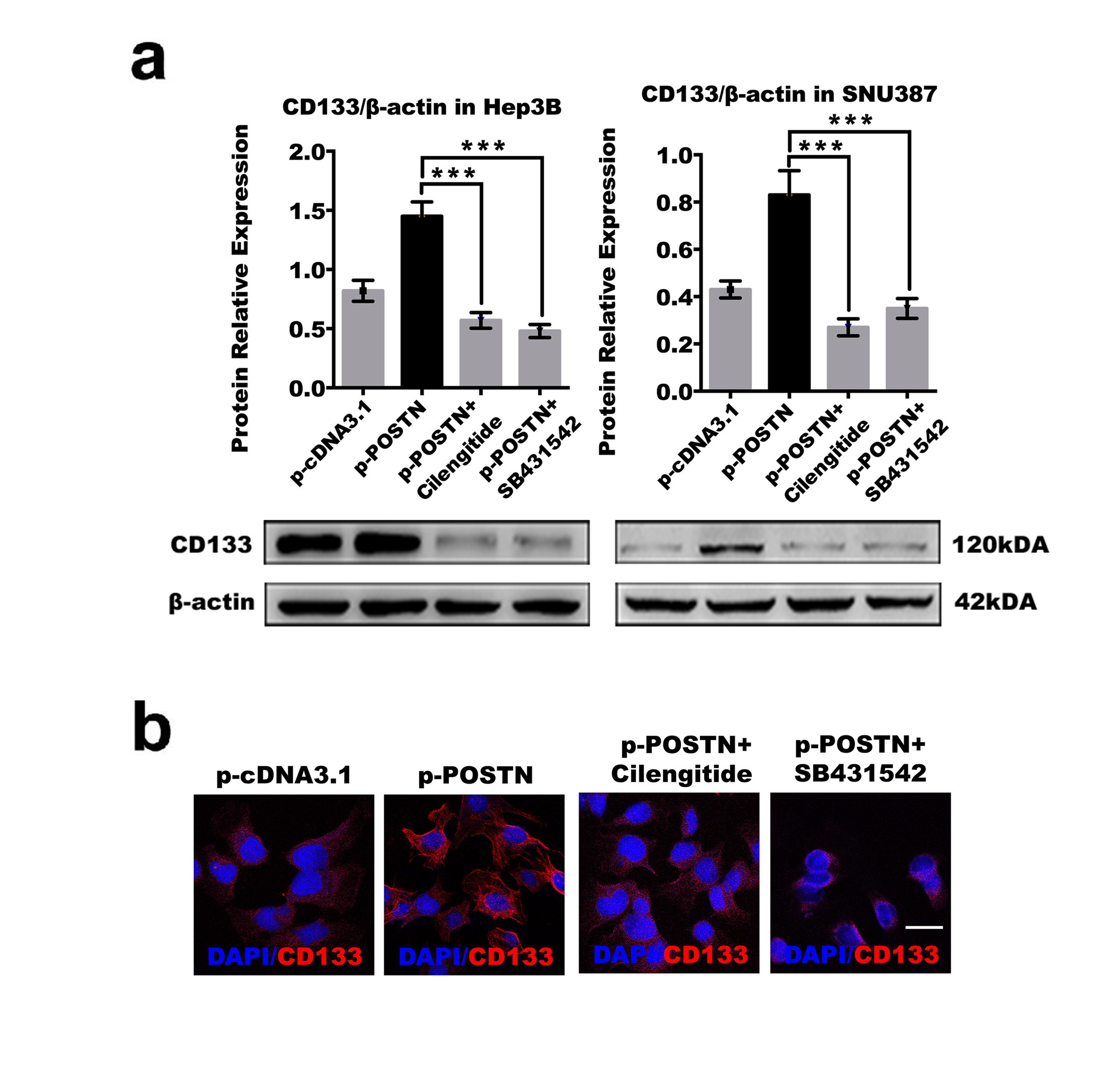

Supplement: Supplementary file 3 — Additional file 3: Fig. S3. (a-b) CD133 protein expression was examined by WB (a) experiment and (b) Immunofluorescence staining after up-regulate POSTN expression, followed by using cilengitide to target αvβ3 and SB431542 to inhibit TGFβ1, ***P < 0.001. [file 13046_2021_2011_MOESM3_ESM.jpg]

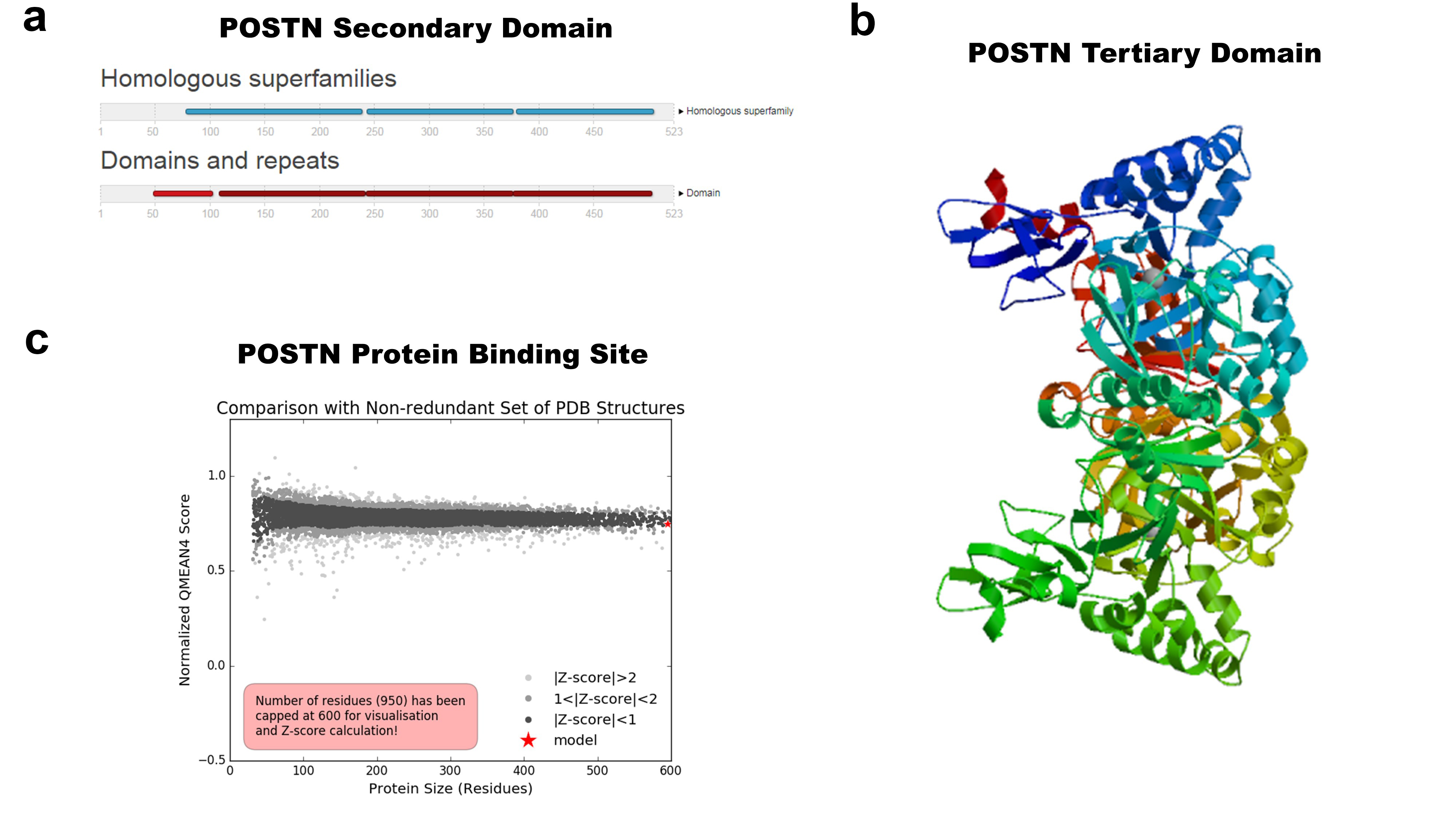

Supplement: Supplementary file 4 — Additional file 4: Fig. S4. Protein structure and binding site of POSTN. [file 13046_2021_2011_MOESM4_ESM.jpg]
