# Supplementary material for: A positive feedback loop between Periostin and TGFβ1 induces and maintains the stemness of hepatocellular carcinoma cells via AP-2α activation
Source: J Exp Clin Cancer Res. 2021 Jun 30;40:218. doi: 10.1186/s13046-021-02011-8 (PMC8243733; doi:10.1186/s13046-021-02011-8)
Supplement: Supplementary file 5 — Additional file 5: Table S1. List of antibodies, reagents and kits used in the study. Table S2. Primer of genes used in this article. [file 13046_2021_2011_MOESM5_ESM.docx]

**Supplementary Table**

**Table S1.** List of antibodies, reagents and kits used in the study

| REAGENTS AND KITS | | |
| --- | --- | --- |
| NAME | CATALOG &PRODUCER | PLACE |
| Cilengitide | HY-16141, Mce | New Jersey, USA |
| lenvatinib | HY-10981, Mce | New Jersey, USA |
| SB431542 | HY-10431, Mce | New Jersey, USA |
| IV collagenase | C5138, sigma | St. Louis, MO,USA |
| diluted elution buffer | 1610747, biorad | Richmond, CA, USA |
| Fetal bovine serum | 10099141, gibco | Grand Island, NY, USA |
| TRIzol | 15596026, Invitrogen | Grand Island, NY, USA |
| RevertAid First Strand cDNA Synthesis Kit | K1621, Thermo Scientific™ | San Jose,CA,USA |
| SYBR Premix Ex Taq | RR420A, Takara | Tokyo, Japan |
| bicinchoninic acid (BCA) kit | 23227, Invitrogen | Grand Island, NY, USA |
| 4%-15% SDS− PAGE gel | biorad | Richmond, CA, USA |
| PVDF membrane | 3010040001, Roche | Basel, Switzerland |
| HyGLO HRP assay kit | 34095, Thermo Scientific™ | San Jose, CA, USA |
| magnetic beads | 1614023, biorad | Richmond, CA, USA |
| Lipofectamine^TM^ 3000 | L3000015, Thermo Scientific™ | San Jose, CA, USA |
| Dual-Glo® Luciferase Assay System | E2920, promega | Madison, Wisconsin, USA |
| Matrigel Invasion Chambers | 354480, corning | Cambridge, MA, USA |
| B27 supplement | 17504044, gibco | Grand Island, NY, USA |
| epidermal growth factor | PHG0311, gibco | Grand Island, NY, USA |
| basic fibroblast growth factor | 13256029, gibco | Grand Island, NY, USA |
| hepatocyte growth factor | 294-HG-025, R&D | Mn, MN, USA |
| methylcellulose | M7027-100G, sigma | St. Louis, MO, USA |
| Cell Counting Kit-8 cell  proliferation assay kit | CK04, dojindo | Kumamoto, USA |

| ANTIBODIES | | | |
| --- | --- | --- | --- |
| Antibodies | Dilution | Source | Address |
| Alexa Fluor 488-conjugated Goat anti-mouse IgG | 1:400 IF | AB150077, ABCAM | Cambridge, MA, USA |
| Alexa Fluor 594-conjugated Goat anti-rabbit IgG | 1:400 IF | AB150080, ABCAM | Cambridge, MA, USA |
| Periostin | 1:1000 WB,  1:200 IF | AB14041, ABCAM | Cambridge, MA, USA |
| Periostin | 25 ug/mL IP | AF3548，NOVUS | Littleton, CO, USA |
| IgG | 500ug/ml IP | sc-2343, Santa cruz | Santa Cruz, CA, USA |
| Goat Anti-Rabbit IgG H&L (HRP) | 1:5000 WB | AB97051, ABCAM | Cambridge, MA, USA |
| Goat Anti-Mouse IgG H&L (HRP) | 1:5000 WB | AB6789, ABCAM | Cambridge, MA, USA |
| Alpha-SMA | 1:500 IHC | 19245, Cell Signaling | Danvers, MA, USA |
| c-Myc | 1:1000 WB | 5605, Cell Signaling | Danvers, MA, USA |
| avβ3 | 1:250 WB, | sc-7312, Santa cruz | Santa Cruz, CA, USA |
| TGFβ1 | 1:100 WB,  1:100 IF | sc-52893, Santa cruz | Santa Cruz, CA, USA |
| β-actin | 1:1000 WB | 4970, Cell Signaling | Danvers, MA, USA |
| GAPDH | 1:1000 WB | 5174, Cell Signaling | Danvers, MA, USA |
| FOXP3 | 1:1000 WB | AB20034, ABCAM | Cambridge, MA, USA |
| CD133 | 1:1000 WB,  1:400 IHC,  1:200 IF, FCM | 64326, Cell Signaling | Danvers, MA, USA |
| AP-2α | 1:1000 WB,  1:100 IF | 3215, Cell Signaling | Danvers, MA, USA |
| CD90 | 1:1000 IHC | 13801, Cell Signaling | Danvers, MA, USA |
| CK19 | 1:500 IHC | ab52625, ABCAM | Cambridge, MA, USA |
| TWIST | 1:500 IHC | ab50581, ABCAM | Cambridge, MA, USA |
| CXCR4 | 1:500 IHC | ab124824, ABCAM | Cambridge, MA, USA |

**Table S2.** Primer of genes used in this article

| Gene Name | Direction | Sequence |
| --- | --- | --- |
| POSTN | FORWARD | CCCCGTGACTGTCTATAAGCC |
| POSTN | REVERSE | TGACCTTGGTGACCTCTTCTTG |
| CD133 | FORWARD | GTGGCGTGTGCGGCTATGAC |
| CD133 | REVERSE | CCAACTCCAACCATGAGGAAGACG |
| AP-2α | FORWARD | GGCGCCGTGACTGGAG |
| AP-2α | REVERSE | TGACGGTCCATGGCTGAAAA |
| C-Myc | FORWARD | TACAACACCCGAGCAAGGAC |
| C-Myc | REVERSE | CGGGAGGCTGGTTTTCCA |
| FOXP3 | FORWARD | GCTGCAGCTCTCAACGGT |
| FOXP3 | REVERSE | TTGAGGGAGAAGACCCCAGT |
